# Ultra-processed food intake and cognitive decline in older adults

**DOI:** 10.1007/s00394-026-03896-x

**Published:** 2026-02-19

**Authors:** Chantal Buis, Mary Nicolaou, Marjolein Visser, Margreet R. Olthof, Hanneke A. H. Wijnhoven

**Affiliations:** 1 Department of Health Sciences, Faculty of Science, Amsterdam Public Health Research Institute, Vrije Universiteit Amsterdam, 1081 HV Amsterdam, The Netherlands; 2 Department of Public and Occupational Health, Amsterdam Public Health Research Institute, Amsterdam University Medical Center, University of Amsterdam, Amsterdam, The Netherlands

**Keywords:** Ultra-processed foods; Cognition; Cognitive decline; Older adults; NOVA classification; Longitudinal study

## Abstract

**Background:**

Global ultra-processed foods (UPFs) intake has increased. While several studies have linked the intake of specific UPF products to cognitive decline, fewer have investigated overall dietary UPF intake, with conflicting results.

**Objective:**

To examine the association of overall UPF intake with cognitive function and 10-y decline among Dutch older adults (≥ 55 years).

**Methods:**

Data from 1371 participants of the Longitudinal Aging Study Amsterdam (LASA) were used.

Cognitive function was assessed four times between 2011/2012 and 2021/2022 using five tests covering global cognition (MMSE), information processing speed (Coding task), episodic memory (15-Word Test) and executive function (Word Fluency and Digit span). Dietary intake was measured in 2014/2015 with a validated food frequency questionnaire. Food items were classified as UPFs based on the NOVA classification. Total UPF intake was expressed as a percentage of total dietary intake in grams, and divided into quartiles (1.5– < 13.2%, 13.2– < 18.5%, 18.5– < 24.9% and 24.9–72.4%). Linear mixed models assessed associations between UPF intake quartiles and cognitive function and decline with age, while adjusting for potential confounders and testing for interaction with sex.

**Results:**

On average, UPFs contributed 20.1% of total dietary intake in grams per day. No associations were found between UPF intake and cognitive function or decline with age for any of the cognitive domains.

**Conclusion:**

We found no evidence of an association between total UPF intake and cognitive function or decline with age in Dutch older adults.

## Introduction

The number of people living with dementia is projected to increase from approximately 57 million globally in 2019 to 153 million by 2050, largely due to population ageing and growth [1]. Dementia is a leading cause of disability and dependency among older adults [2], imposing a substantial burden on individuals, families, and society [3]. As no curative treatment is currently available, identifying modifiable risk factors to prevent or delay dementia is essential [4]. One promising modifiable lifestyle factor that may influence cognitive function and decline, and therefore the development of dementia, is diet [5]. Healthy dietary patterns such as the Mediterranean diet, the Dietary Approaches to Stop Hypertension (DASH) diet and the Mediterranean-DASH diet Intervention for Neurodegenerative Delay (MIND) have been associated with better cognitive function, slower cognitive decline and lower dementia risk, although evidence from randomized controlled trials demonstrated no or small effects of these dietary interventions on cognitive outcomes [6–8].

In addition to healthy dietary patterns, there is increasing attention for the impact of ultra-processed foods [9] as today’s Western dietary pattern is characterized by a high intake of UPFs [10]. UPFs can, according to the NOVA classification, be defined as formulations of food ingredients often modified by chemical processes and assembled into ready-to-consume, hyper-palatable products using cosmetic additives [11]. Examples include ice cream, cookies, sugar-sweetened beverages (SSBs) processed meats, mass-produced packaged breads and buns, and breakfast cereals [11]. UPF intake varies widely between countries, with the highest levels in the United States and United Kingdom (generally > 50% of energy intake) and the lowest in Mediterranean countries such as Italy (approximately 10% of energy intake) [10]. In the Netherlands, older adults obtained about 37% of their energy intake or 18% of their total intake in grams from UPFs in 2015 [12]. Higher UPF intake is clearly linked to poorer diet quality [9] and has been associated with cardiovascular and cerebrovascular disease, type 2 diabetes, chronic kidney disease, Crohn’s disease, depression, obesity and mortality [13, 14]. Additionally, it has been suggested that higher UPF intake may negatively affect cognitive function by altering the gut microbiota, potentially leading to neuroinflammation and neurodegeneration [15].

Several studies have reported associations of the intake of specific UPF products, such as SSBs [16–19] and processed meat [20–23], with poorer cognitive function, cognitive decline or dementia risk, although findings are not consistent across studies [24, 25]. Fewer studies examined overall dietary UPF intake using the NOVA classification, also with mixed findings [23, 26–31]. Some longitudinal studies reported that higher UPF intake was associated with faster cognitive decline [26] and higher risk of Alzheimer’s disease [31], dementia [29] and cognitive impairment [30]. Other cross-sectional [27] and longitudinal [23, 28] studies found no association with cognitive function [27], cognitive decline [23] or the risk of cognitive impairment [28]. Given these inconsistent findings, the aim of the present study is to examine the longitudinal association between overall UPF intake and cognitive function and 10-y decline among Dutch older adults participating in the Longitudinal Aging Study Amsterdam (LASA), using a large battery of cognitive function tests.

## Methods

### Study design and participants

Data were used from the LASA study, which is an ongoing prospective cohort study focusing on the determinants, trajectories and consequences of physical, cognitive, emotional and social functioning of Dutch older adults [32, 33]. The study contains a nationally representative sample of Dutch older adults, who were recruited from three culturally distinct regions in the Netherlands (Amsterdam, Oss and Zwolle). These regions were intentionally selected to capture the major religious and urban–rural variation of the Dutch older population. Participants were randomly drawn from municipal population registers. LASA started in 1992/1993 with a cohort consisting of 3107 participants aged 55 to 85 years old. In 2002/2003, a second cohort and in 2012/2013, a third cohort consisting of 1002 and 1023 Dutch older adults aged 55 to 65 years, respectively, were added to the original sample. Every 3–4 years measurement waves are being conducted. The measurements are performed by trained interviewers, who visited the participants at home and collected data via interviews, self-administered questionnaires and clinical tests. The LASA study was approved by the Medical Ethics Committee of the VU University Medical Center (METC numbers: 92/138, 2002/141, 2012/361, and 2016/301), and all participants provided written informed consent. A detailed description of the sampling and data collection procedures can be found elsewhere [32, 33].

**Fig. 1:**
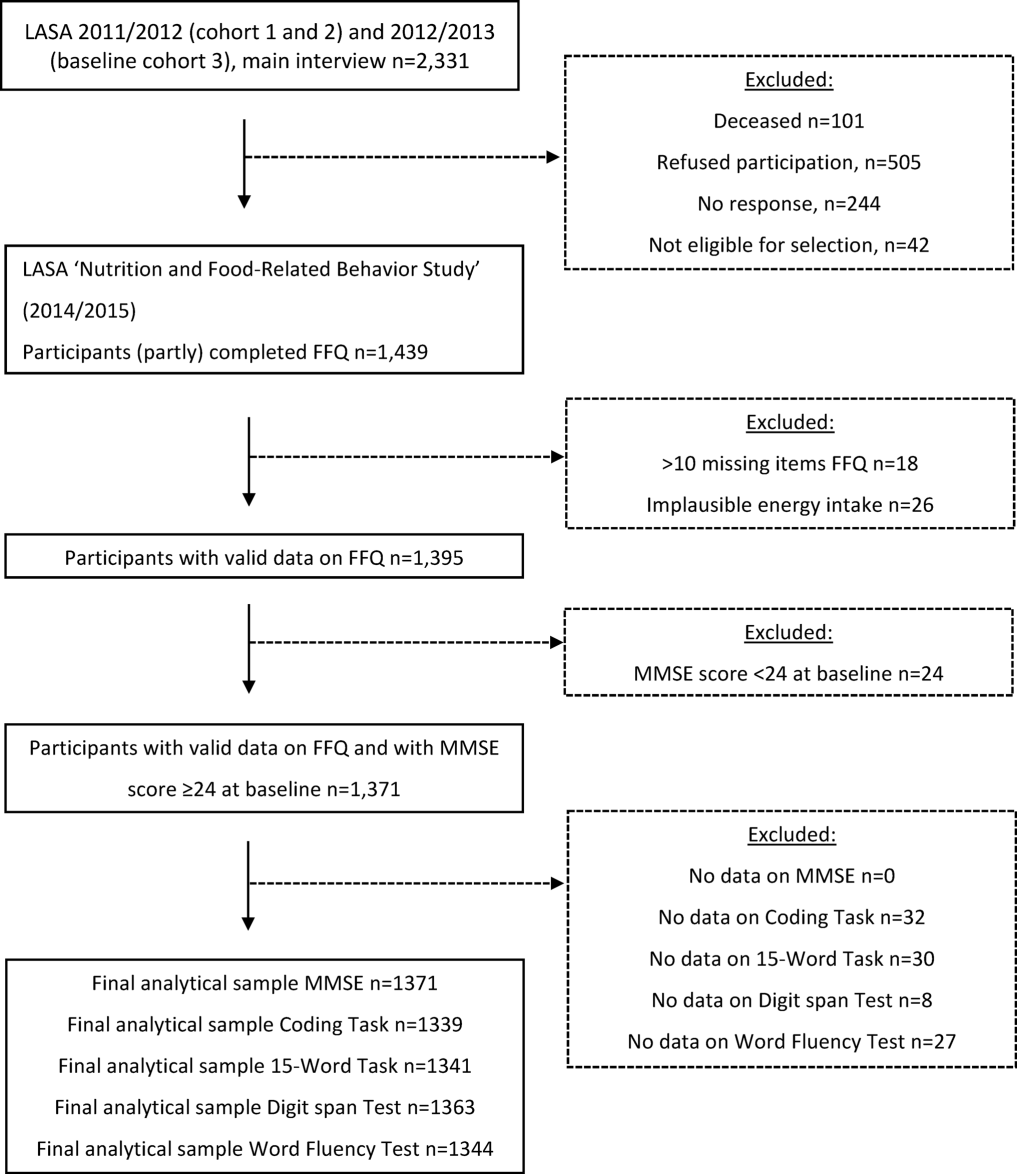
Flowchart with description of the study sample originating from the Longitudinal Aging Study Amsterdam (LASA)

For the present study, participants who completed a Food Frequency Questionnaire (FFQ) as part of the ancillary "LASA Nutrition and Food-Related Behavior Study" which was conducted in 2014/2015 between two regular LASA measurement waves [34, 35], were included. In this ancillary study, a semi-quantitative FFQ was administered to 1439 LASA participants (see Fig. 1 of Winkens et al. [34] for the study flow chart). The 2011/2012 (baseline cohort 1 and 2) and 2012/2013 (baseline cohort 3) measurement waves served as the baseline for cognitive assessments and covariates, with follow-up measurements conducted during the 2015/2016, 2018/2019, and 2021/2022 waves. Of the 1439 participants of the ancillary study, 18 participants with more than 10 missing values on the FFQ questions and 26 participants with implausible energy intake (< 800 kcal or > 4000 kcal for males and < 500 kcal or > 3500 kcal for females [36]) were excluded. Subsequently, 24 participants with a Mini-Mental State Examination (MMSE) score below 24 at baseline in 2011/2013 were excluded to reduce the risk of dietary recall bias due to cognitive impairment, as such scores are indicative of dementia [37]. For each cognitive test, including the MMSE, the Coding Task, the 15-Word Task (15WT), the Digit Span Test, and the Word Fluency Test, participants without any available data (at baseline and follow-up) were excluded. The final analytical samples consisted of 1,371 participants for the MMSE, 1,339 for the Coding Task, 1,341 for the 15WT, 1,363 for the Digit Span Test, and 1,344 for the Word Fluency Test. A flowchart of the study sample is presented in Fig. 1.

### Measurements

#### UPF intake (NOVA classification)

Dietary intake was evaluated using a 238-item semi-quantitative FFQ, which captured habitual intake over the preceding four weeks. This FFQ, adapted and validated for use in the Dutch population, was originally developed for the Healthy Life in an Urban Setting (HELIUS) study [38]. Its relative validity was examined in a subsample of 88 older LASA participants (mean age 71.9 years) who also completed three 24-h dietary recalls [35]. Results indicated a group-level bias within ± 5% for energy and macronutrient intake, with Pearson correlation coefficients ranging from 0.26 to 0.72 and moderate to high agreement across quintiles. For most micronutrients and food groups, validity was moderate (r = 0.3–0.5), although lower correlations were observed for β-carotene, vitamin B1, fish, and grains [24]. These validity outcomes are consistent with those typically reported for FFQs and are considered acceptable in cohort studies using dietary assessments [39].

Daily intake of each food and beverage item (hereafter referred to as ‘food’) was estimated in grams per day by multiplying reported portion sizes by intake frequency. Each food was subsequently linked to the relevant codes from the Dutch Food Composition Database (In Dutch: NEVO; “Nederlands Voedingsstoffenbestand”), which provides standardized nutritional information for foods commonly consumed in the Netherlands [40]. Each food consisted of several underlying items which were weighted according to their consumption by the Dutch population, based on national food consumption data [38]. For example, the food ‘candy bar’ was linked to seven specific NEVO codes (Mars, Twix, Snickers, Milky Way, KitKat, Lion, and Bounty) with corresponding consumption frequencies of 33.3%, 15.6%, 14.7%, 12.0%, 9.0%, 7.8%, and 7.6%, respectively.

To categorize each food as ultra-processed or not, we applied the NOVA classification system, which groups all foods into four categories based on the degree of processing [11]. NOVA group 1 includes unprocessed and minimally processed foods, such as fresh, dried or frozen vegetables and fruits. Group 2 includes processed culinary ingredients, like molasses and sugar obtained from beet or cane. Group 3 includes processed foods, such as sugared or salted seeds and nuts. Group 4 includes UPFs, like cookies and ice cream. Two researchers independently classified all food and beverage items included in the FFQ based on their level of processing. When uncertainty arose regarding the classification of a specific item, ingredient lists were checked using websites of Dutch supermarkets. Any remaining ambiguities were resolved through additional literature searches and discussion between the researchers.

Next, we calculated, for each FFQ food, the percentage of its underlying NEVO components that were classified as ultra-processed (NOVA group 4). For example, food categorized as UPFs with only one corresponding NEVO code, such as ‘spring roll prepared’ and ‘milk chocolate-full’, were composed of 100% UPF. For food with multiple NEVO codes, we used the weighting as mentioned above to calculate the proportion of the food as UPF. For instance, the FFQ food ‘candybars’, was composed of 100% UPF, because all seven corresponding NEVO codes were classified as UPF. The FFQ food ‘grains for porridge’ was composed of four NEVO codes, including breakfast product ‘Brinta’, ‘meal oat’, ‘breakfast product Albona 7-grains-energy breakfast’ and ‘Flour rice-Bambix’. The corresponding frequency of intake in the Dutch population of these items was 79.7%, 16.4%, 2.3% and 1.6%, respectively. Given that only ‘Flour-rice Bambix’ was classified as UPF, the FFQ food ‘grains for porridge’ was composed of 1.6% UPF.

For each participant, the UPF proportion of each FFQ food was multiplied by the participant’s reported daily intake of that food in grams to calculate the daily intake of UPFs in grams per food. The daily intake of UPFs from all FFQ foods was then summed to determine each participant’s total daily UPF intake in grams. For the present study, overall daily UPF intake was expressed as a percentage of the participant’s total daily intake of foods in grams, with a higher percentage indicating a higher contribution of UPFs to the diet. We used a weight-based rather than energy-based approach to capture non-nutritional components related to food processing, such as additives, and to include UPFs that do not provide energy, such as artificially sweetened beverages [41]. Alcoholic beverages were not included as UPFs, and were excluded from the weight ratio, because alcohol intake is associated with both higher UPF intake and adverse cognitive outcomes [42–44]. The association between alcohol intake and adverse cognitive outcomes [42, 44] is likely due to the neurotoxic effects of the ethanol molecule itself or the nutritional deficiencies related to alcohol use [45]. We therefore decided to include alcohol intake as a confounding factor.

#### Cognitive function

The dependent variable, cognitive function, was repeatedly measured during the regular LASA measurement waves. Cognitive function was assessed in four domains using five tests: global cognition (MMSE), information processing speed (Coding Task), episodic memory (15-Word Test), and executive function (Word Fluency and Digit Span).

The MMSE is a frequently used screening instrument for global cognitive impairment [46] and was used to measure global cognition. The total MMSE score ranges from 0 to 30 with a higher score indicating better cognitive functioning.

The Coding Task was used to measure information processing speed. The Coding Task used is an adapted version of the Alphabet Coding Task-15 [47]. In this timed letter-symbol substitution task, participants verbally indicated the matching symbol for each letter across three one-minute trials. Verbal instead of written responses were collected to minimize delays from writing and to prevent difficulties in interpreting handwriting during scoring [48]. The mean score on the three trials was used, which ranged from 8.0 to 47.7. A higher score indicates faster information processing speed.

The 15WT was used to measure episodic memory. This test is derived from the Auditory Verbal Learning Test [49]. Participants had to learn 15 words during three trials, instead of the original five trials due to limited interview time, and were asked to recall as many words as possible immediately after every trial and after a 20-min delay. The mean score (range 0–25) of the following three scores was used: the total score on the three trials for immediate recall (range 0–45), the maximum score on the three trials for immediate recall (ranged 0–15) and the total score on the delayed recall (range 0–15). A higher score indicates better episodic memory.

The Digit span test was used to measure executive functioning. This test is a subtest of the Wechsler Adult Intelligence Scale [50]. Participants were asked to recall a set of numbers forwards and backwards whereby the sequence-length of the numbers increased. The total number of correct sets of numbers forwards and backwards was used. The total score ranged from 0 to 30, with a higher score indicating better executive functioning.

The Word Fluency Test, which is a verbal fluency test, was also used to measure executive functioning. During the Word Fluency Test participants were asked to name as many words as possible that begin with the letter D in one minute, which assessed phonemic fluency. The score of this task ranged from 0 to 36. After that, participants were asked to name as many animals as possible in one minute, which assessed semantic fluency. The score of this task ranged from 1 to 40. The mean score of the two tasks was used, and a higher score indicated better executive functioning.

All test scores were standardized into z-scores by using the sample mean and standard deviation (SD) of each test at baseline to be able to compare the different test scores. For tests that consisted of multiple components (15WT and Word Fluency Test), the mean of the individual z-scores was used in the analyses.

#### Covariates

During the first main LASA interview, data were collected on: sex at birth (male/female), birth date in order to calculate age (years) at each measurement wave, and the highest level of education completed by the participant. Education was categorized into low (not completed elementary and completed elementary), middle (lower vocational, general intermediate, intermediate vocational and general secondary education) and high (higher vocational education, college and university). During each measurement cycle, data were collected about partner status, body weight and height, physical activity, alcohol intake, smoking status, depressive symptoms and presence of chronic disease. Partner status was determined by a closed question with the following answer options: no partner, partner co-residing and partner residing outside the household. No partner and partner residing outside the household were categorized into living alone, while partner co-residing was categorized into living with a partner. BMI was calculated by dividing measured body weight in kilograms (kg) by measured body height in meters (m) squared. Body weight was measured to the nearest 0.1 kg using a calibrated bathroom scale (Seca, model 100, Lameris, Utrecht, the Netherlands) and body height was measured to the nearest 0.001 m using a stadiometer. Physical activity was assessed using the LASA Physical Activity Questionnaire (LAPAQ) [51], in which participants were asked about frequency and duration of walking outdoors, biking, gardening, light and heavy household activities and sports in the last 2 weeks. Physical activity was defined as total metabolic equivalent (MET) hours per week spent on all these activities. Alcohol intake was assessed by asking the participants whether they drank alcoholic beverages. If so, they were asked how many days per week they drank these beverages and the number each day. Participants were classified into the following four categories using the alcohol intake index developed by Garretsen [52]: does not drink (0 days/month), light drinking (6 or more drinks on < 1 day/month, 4–5 drinks on < 4 days/month, 2–3 drinks on < 3 days/week, 0–1 drinks/day), moderate drinking (6 or more drinks on 1–3 days/month, or 4–5 drinks on 1–4 days/week, or 2–3 drinks on 3–7 days/week) and (very) excessive drinking (6 or more consumptions on 1–7 days/week or 4–5 drinks on 5–7 days/week). Smoking status was asked and divided into never, former and current smoker. Depressive symptoms were measured with the Center for Epidemiologic Studies Depression Scale (CES-D) [53], which is a self-reported symptom rating scale. The total score ranged from 0 to 60, with a higher score representing a higher level of depressive symptoms. The presence of chronic diseases was self-reported and defined as the number of chronic diseases from frequently occurring somatic chronic diseases in the Netherlands, including chronic non-specific lung disease, cardiac disease, peripheral arterial disease, diabetes, cerebrovascular accident or stroke, arthritis and cancer [54]. The total score ranged from 0 to 7. This score was categorized into no chronic disease, one chronic disease and two or more chronic diseases. Finally, total energy intake (kcal/day) and diet quality were based on the FFQ assessed during LASA ancillary study. Diet quality was defined as adherence to the Dutch Healthy Diet index 2015 (DHD15-index) [55]. The index is used to assess adherence to the Dutch dietary guidelines [56], includes 13 components (vegetables, fruits, whole grain products, legumes, nuts and seeds, dairy, fish, tea, fats and oils, red meat, processed meat, sugar-sweetened beverages and fruit juices, alcohol) instead of the original 15 components. Information on salt and type of coffee consumption was unavailable, so these were excluded. Each component was scored from 0 to 10, giving a total range of 0 (no adherence) to 130 (full adherence) [55].

### Statistical analyses

Descriptive statistics were performed to characterize the total study sample (comprised of participants with an MMSE baseline measurement) and stratified by quartiles of overall UPF intake. Continuous variables were presented as means with standard deviation (± SD) when normally distributed or as medians with interquartile range (IQR) when not normally distributed. Categorical variables were summarized as frequencies and percentages.

Linear mixed model analyses were used to examine the association between quartiles of overall UPF intake (%) and repeated measures of Z scores for the various cognitive measures. UPF intake, expressed as a percentage of total daily intake in grams, was categorized into quartiles consistent with previous studies [23, 26, 29], which resulted in the following categories: 1.5–13.2%, 13.2–18.5%, 18.5–24.9% and 24.9–72.4%. Quartiles were used to allow comparison with previous literature and to capture potential non-linear associations. Following earlier approaches [57, 58], linear mixed model analyses were used to model both level and change in cognitive function by quartiles of overall UPF intake with the first quartile used as the reference. The level models tests the association between UPF intake and cognitive function over time, thus capturing both cross-sectional and longitudinal associations. Age and age^2^ at each measurement, along with other covariates, were included in the model: Cognition​ = Intercept + UPF quartile ​ + Age ​ + Age^2^​ + other covariates + Random effects + Error. To assess the association between UPF intake and cognitive decline with aging, change models were used in which an interaction term between UPF quartiles and age at each measurement was included: Cognition = Intercept + UPF quartile + Age + (UPF quartile × Age) + Age^2^ + Other covariates + Random effects + Error. This interaction term assessed whether the rate of cognitive change varied by UPF intake during aging. In a previous study using the same approach [58], we performed a sensitivity analyses to assess the robustness of our findings by including time of assessment instead of age at each measurement for the change models, while adjusting for baseline age and baseline age^2^, to allow an estimation of the rate of cognitive decline per study wave, rather than per age year. As this yielded the same results, we did not perform this sensitivity analysis again. Models included a random intercept for repeated measurements and a random slope for age when it improved model fit (included in all models except for the Word Fluency Test). Improvement in model fit was evaluated using likelihood ratio test with an unstructured covariance matrix for the random effects. Z scores were analyzed continuously and model assumptions were verified and met via analyses of residuals using histograms, Q-Q plots and scatterplots. All available data were included, as mixed-effects models accommodate missing values.

For all analyses, three hierarchical models were fitted. Model 1 adjusted for age at each measurement, age squared [57], sex and education. Model 2 additionally adjusted for partner status, total energy intake, BMI, physical activity, alcohol intake, smoking status, depressive symptoms and presence of chronic diseases. Model 3 further adjusted for diet quality. Time-independent covariates included sex, education, total energy intake and diet quality; time-dependent covariates included age, partner status, BMI, physical activity, alcohol intake, smoking status, depressive symptoms and presence of chronic diseases. In the fully adjusted models, interaction with sex was tested, as prior research suggested potential sex differences in the association between UPF intake and cognitive decline, with some associations only observed in females [23]. Interaction between quartiles of UPF intake and sex was tested in the fully adjusted level models, while in the fully adjusted change models three-way interaction between quartiles of UPF intake, sex and age was tested. If at least one of the interaction terms in the hierarchical models was statistically significant (*p*-value < 0.05), all three adjustment models for that specific cognitive test were stratified by sex, while analyses for the other cognitive tests and model types (level or change) were not stratified. To visualize cognitive trajectories across UPF intake quintiles, predicted values from the fully adjusted change models, including the interaction with age, were plotted against age. Age and age^2^ were centered at 55 years since that is the starting age of LASA cohort. Only the lowest and highest quintiles were depicted, illustrating contrasts in cognitive function and age-related decline associated with lowest versus highest UPF intakes for the sample.

One sensitivity analysis was performed using the fully adjusted model, by creating an additional UPF weight ratio in which bread was excluded to test the robustness of the results. It is difficult to classify breads as either UPF or not according to NOVA [11], as the exact interpretation of the terminology used, such as mass-produced, is not self-evident [59]. Moreover, a previous study using data from the UK Biobank found that high bread intake was associated with decreased risk of dementia [60]. In addition, Dutch people consume a lot of bread [61].

All statistical analyses were performed using SPSS Statistics (version 28, IBM Corp, Armonk, NY, USA) for descriptive statistics and Stata Statistical Software (release 17, StataCorp LLC, College Station, Texas, USA) for linear mixed models. For all analyses, a two-sided p-value of < 0.05 was considered statistically significant.

## Results

Baseline characteristics of the total study sample and by quartiles of overall UPF intake are shown in Table 1. The mean age of participants was 67.3 ± 8.2 years, 722 participants (52.7%) were female and 162 (11.8%) had a low educational level. On average, UPFs contributed 20.1% of total intake in grams per day. Compared with those in lowest quartile of overall UPF intake, participants in the highest quartile were less often female (33.3 vs 70.8%) and less often had a high educational level (20.8 vs 35.1%). They also had a higher mean total energy intake (2338.4 vs 1741.2 kcal/day), lower mean diet quality (75.1 vs 88.1) and lower median physical activity levels (47.3 vs 57.5 MET hours/week) compared to participants in the lowest quartile.

**Table 1:** Baseline characteristics of total study sample of Dutch older adults participating in the Longitudinal Aging Study Amsterdam and by quartiles of overall UPF intake

	Total n = 1371	Q1 (1.5–13.2%) n = 342	Q2 (13.2–18.5%) n = 343	Q3 (18.5–24.9%) n = 344	Q4 (24.9–72.4%) n = 342
Age (years)	67.3 ± 8.2	66.4 ± 7.6	66.9 ± 7.8	68.1 ± 8.3	67.5 ± 8.9
Female	722 (52.7)	242 (70.8)	208 (60.6)	158 (45.9)	114 (33.3)
Education					
Low	162 (11.8)	36 (10.5)	35 (10.2)	40 (11.6)	51 (14.9)
Middle	801 (58.4)	186 (54.4)	187 (54.5)	208 (60.5)	220 (64.3)
High	408 (29.8)	120 (35.1)	121 (35.3)	96 (27.9)	71 (20.8)
Living alone	347 (25.3)	97 (28.4)	89 (25.9)	79 (23.0)	82 (24.0)
Total energy intake (kcal/day)	2078.3 ± 575.4	1741.2 ± 456.4	2026.7 ± 500.5	2206.4 ± 562.1	2338.4 ± 593.2
DHD15-index score^a^	82.4 ± 16.0	88.1 ± 15.3	84.4 ± 14.9	81.9 ± 15.7	75.1 ± 15.2
BMI (kg/m^2^)*	27.3 ± 4.2	27.3 ± 4.3	27.0 ± 4.2	27.0 ± 3.8	27.8 ± 4.7
Physical activity (MET hours/week)*	53.5 (33.1–79.5)	57.5 (36.5–82.8)	55.5 (34.2–78.9)	51.9 (32.4–81.3)	47.3 (27.9–73.9)
Alcohol intake*					
Does not drink	163 (12.3)	40 (12.2)	35 (10.6)	33 (9.9)	55 (16.5)
Light drinking	625 (47.3)	147 (45.0)	152 (46.2)	160 (48.0)	166 (49.8)
Moderate drinking	455 (34.4)	125 (38.2)	120 (36.5)	122 (36.6)	88 (26.4)
(Very) excessive drinking	79 (6.0)	15 (4.6)	22 (6.7)	18 (5.4)	24 (7.2)
Smoking status*					
Never	371 (28.1)	98 (30.1)	104 (31.5)	82 (24.6)	87 (26.1)
Former	796 (60.2)	194 (59.5)	191 (57.9)	206 (61.9)	205 (61.6)
Current	155 (11.7)	34 (10.4)	35 (10.6)	45 (13.5)	41 (12.3)
Depressive symptoms^b^,*	5.0 (2.0–10.0)	5.0 (2.0–10.0)	6.0 (2.0–10.0)	5.0 (2.0–9.0)	5.0 (2.0–10.0)
Chronic diseases					
No	421 (30.7)	94 (27.5)	104 (30.3)	122 (35.5)	101 (29.5)
One	542 (39.5)	156 (45.6)	138 (40.2)	115 (33.4)	133 (38.9)
Two or more	408 (29.8)	92 (26.9)	101 (29.4)	107 (31.1)	108 (31.6)
Total UPF intake (% of total intake in g/day)	20.1 ± 9.7	9.9 ± 2.3	15.7 ± 1.5	21.3 ± 1.8	33.3 ± 8.2
Cognitive function scores^c^					
Global cognition (MMSE)	29 (28–30)	29 (28–30)	29 (28–30)	29 (28–29)	29 (27–29)
Information processing speed (Coding Task) n = 1302	29.4 ± 6.2	30.4 ± 6.3	29.6 ± 6.0	29.0 ± 6.5	28.5 ± 6.1
Episodic memory (15WT) n = 1306	13.6 ± 3.6	14.1 ± 3.5	14.1 ± 3.4	13.3 ± 3.5	12.7 ± 3.6
Executive function (Digit span test) n = 1343	14.2 ± 3.4	14.3 ± 3.3	14.5 ± 3.4	14.0 ± 3.4	13.8 ± 3.4
Executive function (Word Fluency Test) n = 1312	16.9 ± 4.4	17.3 ± 4.5	17.4 ± 4.5	16.7 ± 4.4	16.3 ± 4.3

MMSE, Mini-Mental State Examination; UPF, ultra-processed food; Q, quartile; DHD15-index, Dutch Healthy Diet index 2015; BMI, body mass index; MET, metabolic equivalent; 15WT, 15-Word Learning Task

Normally distributed continuous variables are presented as means ± standard deviation, non-normally distributed continuous variables are presented as medians with interquartile range (25–75th percentile) and categorical variables are presented as n (%)

Overall UPF intake is presented as percentage of total food/beverage intake in grams per day categorized into quartiles

^a^Dutch Healthy Diet index 2015 score ranged from 0 to 130, with a higher score indicating higher adherence and therefore better diet quality [55]

^b^Measured with the Center for Epidemiologic Studies Depression Scale [53]. The total score ranged from 0 to 60, with a higher score indicating a higher level of depressive symptoms

^c^A higher score indicates better cognitive function

*Variable with missing data (< 5%)

**Fig. 2:**
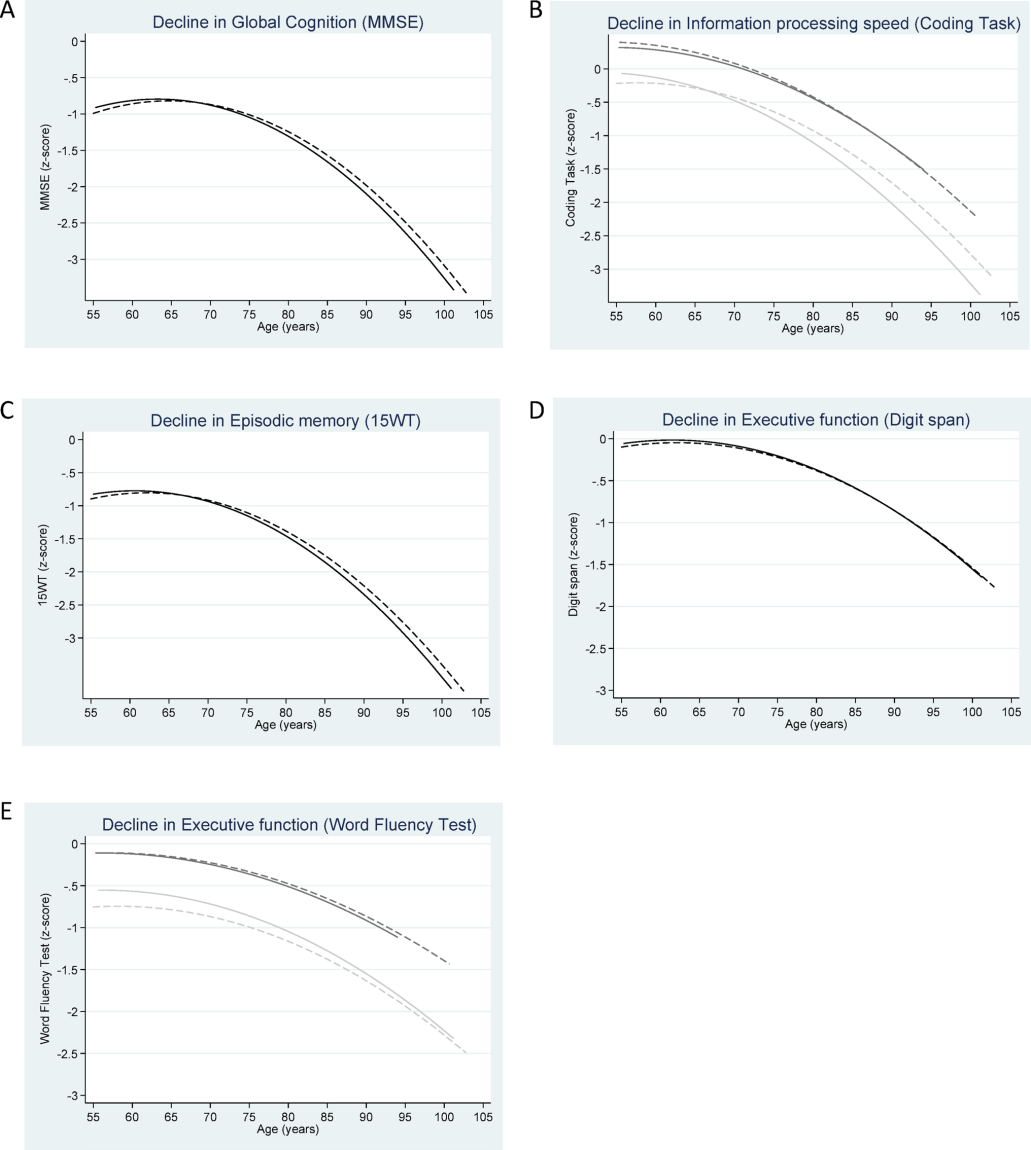
Cognitive domains (z-scores) as a function of calendar age by overall ultra-processed food intake (lowest quartile (1.5–13.2%) (solid line) vs highest quartile (24.9–72.4%) (dashed line)) among Dutch older adults participating in the Longitudinal Aging Study Amsterdam. Adjusted for age, age^2^, sex, education, partner status, total energy intake, BMI, physical activity, alcohol intake, smoking status, depressive symptoms, presence of chronic diseases and diet quality. In plots stratified for sex (B and E), the light gray line represents males and the dark gray line represents females. MMSE, Mini-Mental State Examination; 15WT, 15-Word Learning Task

Results of the linear mixed model analyses of cognitive function level and change during aging by quartiles of overall UPF intake are presented in Table 2. Figure 2 visualizes fully adjusted cognitive trajectories for the first and fourth quartiles of UPF intake over the examined age range. None of the associations between UPF intake and level of cognitive function was statistically significant. A statistically significant interaction between sex and UPF intake was observed for level of executive function measured with the Word Fluency Test (*p* = 0.04 for both the third and fourth quartiles). However, in the stratified analyses, associations for third quartile versus the first quartile were not statistically significant for either males (quartile 3 vs. quartile 1: β = − 0.14 (− 0.32, 0.03); quartile 4 vs. quartile 1: β = − 0.14 (− 0.32, 0.04) or females (quartile 3 vs quartile 1: β = 0.03 (− 0.11, 0.17); quartile 4 vs. quartile 1: β = 0.02 (− 0.14, 0.19).

**Table 2:** Level and change in cognitive function domains by quartiles of overall ultra-processed food intake among Dutch older adults participating in the Longitudinal Aging Study Amsterdam

Domain			Model 1^a^ β (95% CI)	Model 2^b^ β (95% CI)	Model 3^c^ β (95% CI)	Sensitivity analysis^d^ β (95% CI)
Global cognition (MMSE) n = 1371	Level	Q2	− 0.05 (− 0.17, 0.07)	− 0.01 (− 0.13, 0.11)	− 0.01 (− 0.13, 0.12)	0.05 (− 0.07, 0.17)
		Q3	− 0.08 (− 0.21, 0.04)	− 0.06 (− 0.19, 0.07)	− 0.05 (− 0.18, 0.08)	− 0.08 (− 0.21, 0.05)
		Q4	− 0.08 (− 0.21, 0.05)	− 0.02 (− 0.15, 0.12)	− 0.00 (− 0.14, 0.14)	0.04 (− 0.09, 0.18)
	Change	Q2 × age	− 0.00 (− 0.02, 0.01)	− 0.00 (− 0.02, 0.01)	− 0.00 (− 0.02, 0.01)	− 0.00 (− 0.02, 0.01)
		Q3 × age	0.01 (− 0.00, 0.03)	0.01 (− 0.00, 0.03)	0.01 (− 0.00, 0.03)	0.01 (− 0.00, 0.03)
		Q4 × age	0.01 (− 0.01, 0.02)	0.01 (− 0.01, 0.02)	0.01 (− 0.01, 0.02)	0.00 (− 0.01, 0.02)
Information processing speed (Coding Task) n = 1339	Level	Q2	− 0.05 (− 0.18, 0.08)	− 0.04 (− 0.17, 0.09)	− 0.03 (− 0.17, 0.10)	− 0.03 (− 0.16, 0.10)
		Q3	− 0.05 (− 0.18, 0.08)	− 0.04 (− 0.17, 0.10)	− 0.03 (− 0.17, 0.10)	− 0.10 (− 0.24, 0.04)
		Q4	− 0.00 (− 0.14, 0.14)	0.03 (− 0.11, 0.18)	0.05 (− 0.10, 0.19)	0.04 (− 0.11, 0.18)
	Change (male)	Q2 × age	0.01 (− 0.01, 0.03)	0.01 (− 0.00, 0.03)	0.01 (− 0.00, 0.03)	− 0.00 (− 0.02, 0.01)
		Q3 × age	0.01 (− 0.00, 0.03)	0.02 (− 0.00, 0.03)	0.02 (− 0.00, 0.03)	0.01 (− 0.01, 0.02)
		Q4 × age	0.01 (− 0.00, 0.03)	0.01 (− 0.00, 0.03)	0.01 (− 0.00, 0.03)	− 0.00 (− 0.01, 0.01)
	Change (female)	Q2 × age	0.01 (− 0.00, 0.02)	0.01 (− 0.00, 0.02)	0.01 (− 0.00, 0.02)	0.00 (− 0.01, 0.01)
		Q3 × age	− 0.01 (− 0.02, 0.01)	− 0.01 (− 0.02, 0.01)	− 0.01 (− 0.02, 0.01)	− 0.00 (− 0.02, 0.01)
		Q4 × age	− 0.00 (− 0.02, 0.01)	− 0.00 (− 0.02, 0.01)	− 0.00 (− 0.02, 0.01)	− 0.01 (− 0.02, 0.01)
Episodic memory (15WT) n = 1341	Level	Q2	− 0.01 (− 0.12, 0.11)	− 0.01 (− 0.13, 0.11)	0.01 (− 0.11, 0.13)	− 0.01 (− 0.13, 0.10)
		Q3	− 0.05 (− 0.16, 0.07)	− 0.05 (− 0.17, 0.07)	− 0.03 (− 0.15, 0.10)	− 0.06 (− 0.18, 0.06)
		Q4	− 0.05 (− 0.17, 0.08)	− 0.03 (− 0.16, 0.10)	0.02 (− 0.11, 0.15)	0.06 (− 0.07, 0.19)
	Change	Q2 × age	− 0.00 (− 0.02, 0.01)	− 0.00 (− 0.01, 0.01)	− 0.00 (− 0.01, 0.01)	0.01 (− 0.00, 0.02)
		Q3 × age	0.00 (− 0.01, 0.01)	0.01 (− 0.01, 0.02)	0.01 (− 0.01, 0.02)	0.01 (− 0.00, 0.02)
		Q4 × age	0.01 (− 0.00, 0.02)	0.01 (− 0.01, 0.02)	0.01 (− 0.01, 0.02)	0.01 (− 0.00, 0.02)
Executive function (Digit span test) n = 1363	Level	Q2	0.02 (− 0.14, 0.17)	0.01 (− 0.12, 0.14)	0.01 (− 0.12, 0.15)	0.05 (− 0.09, 0.18)
		Q3	0.01 (− 0.14, 0.17)	− 0.02 (− 0.16, 0.12)	− 0.01 (− 0.15, 0.12)	− 0.01 (− 0.14, 0.13)
		Q4	− 0.08 (− 0.25, 0.08)	− 0.03 (− 0.17, 0.12)	− 0.02 (− 0.17, 0.13)	0.06 (− 0.09, 0.21)
	Change	Q2 × age	0.00 (− 0.02, 0.02)	− 0.01 (− 0.02, 0.00)	− 0.01 (− 0.02, 0.00)	− **0.01 (**− **0.02, **− **0.00)**
		Q3 × age	0.02 (− 0.00, 0.03)	0.00 (− 0.01, 0.01)	0.00 (− 0.01, 0.01)	− 0.00 (− 0.01, 0.01)
		Q4 × age	0.02 (− 0.00, 0.03)	0.00 (− 0.01, 0.01)	0.00 (− 0.01, 0.01)	− 0.00 (− 0.01, 0.01)
Executive function (Word Fluency Test) n = 1344	Level (male)	Q2	− 0.12 (− 0.30, 0.06)	− 0.15 (− 0.33, 0.03)	− 0.13 (− 0.31, 0.06)	0.02 (− 0.16, 0.20)
		Q3	− 0.11 (− 0.29, 0.06)	− 0.16 (− 0.34, 0.02)	− 0.14 (− 0.32, 0.03)	− 0.08 (− 0.26, 0.09)
		Q4	− 0.14 (− 0.31, 0.03)	− 0.17 (− 0.35, 0.00)	− 0.14 (− 0.32, 0.04)	− 0.01 (− 0.19, 0.16)
	Level (female)	Q2	0.08 (− 0.04, 0.21)	0.03 (− 0.09, 0.16)	0.04 (− 0.09, 0.17)	− 0.00 (− 0.13, 0.12)
		Q3	0.10 (− 0.03, 0.24)	0.02 (− 0.12, 0.16)	0.03 (− 0.11, 0.17)	− 0.05 (− 0.19, 0.09)
		Q4	0.06 (− 0.09, 0.21)	− 0.00 (− 0.16, 0.16)	0.02 (− 0.14, 0.19)	0.00 (− 0.16, 0.16)
	Change	Q2 × age	− 0.01 (− 0.02, 0.00)	− 0.01 (− 0.02, 0.00)	− 0.01 (− 0.02, 0.00)	− 0.00 (− 0.01, 0.01)
		Q3 × age	0.01 (− 0.00, 0.02)	0.01 (− 0.00, 0.02)	0.01 (− 0.00, 0.02)	0.00 (− 0.01, 0.01)
		Q4 × age	0.00 (− 0.01, 0.01)	− 0.00 (− 0.01, 0.01)	− 0.00 (− 0.01, 0.01)	− 0.00 (− 0.01, 0.01)

β = standardized regression coefficient; CI = confidence interval; MMSE = Mini-Mental State Examination; Q = quartile; 15WT = 15-Word Learning Task

Overall ultra-processed food intake is defined as percentage of total food/beverage intake in grams per day categorized into quartiles (1.5–13.2%, 13.2–18.5%, 18.5–24.9% and 24.9–72.4%)

Values are calculated compared to cognitive function in the first quartile by using linear mixed model analyses with random intercept and random slope for age (if it improved model fit). Negative values indicate worse cognitive function or faster cognitive decline compared to the first quartile. Values in bold denote a p-value < 0.05

^a^Model 1: Adjusted for age, age^2^, sex and education

^b^Model 2: Model 1 + partner status, total energy intake, Body Mass Index, physical activity, alcohol intake, smoking status, depressive symptoms and presence of chronic diseases

^c^Model 3: Model 2 + diet quality (Dutch Healthy Diet index 2015)

^d^Sensitivity analysis excluding bread from the ultra-processed food weight ratio, based on third model (fully adjusted model)

None of the associations between UPF intake and cognitive decline reached statistical significance. A statistically significant interaction between sex and UPF intake was observed for change during aging in information processing speed (*p* = 0.03 for the third quartile). However, in the stratified analyses, associations for third quartile versus the first quartile were not statistically significant for either males (β = 0.02 (− 0.00, 0.03)) or females (β = − 0.01 (− 0.02, 0.01)).

The sensitivity analysis, in which bread was excluded from the UPF weight ratio, yielded results largely consistent with the main analyses (Table 2). The only exception was the association between the second quartile of UPF intake and change in executive function (Digit Span Test), which became statistically significant; the negative beta (β = − 0.01, 95% CI − 0.02, 0.00) indicates a faster decline in executive function in the second compared to the first quartile.

## Discussion

This study aimed to examine the longitudinal association between overall UPF intake and cognitive function and decline among Dutch older adults participating in the Longitudinal Aging Study Amsterdam. The findings show that total UPF intake was not associated with either the level of cognitive function or with cognitive decline with aging for any of the assessed domains, including global cognition, information processing speed, episodic memory, or executive function.

Previous studies investigating the association between overall UPF intake and cognitive function and decline have yielded inconsistent results, despite all using the NOVA classification system similar to the present study [23, 26–31]. Our findings are consistent with two recent longitudinal studies [23, 28] and a cross-sectional study [27] reporting no association between total UPF intake and cognitive function level or decline. In an Israeli cohort of adults aged ≥ 65 years with type 2 diabetes (n = 568), total UPF intake was not associated with decline in global cognition, episodic memory, attention/working memory, semantic categorization/language and executive function [23]. Similarly, a study of middle-aged and older adults in the United States (n = 4750) found no association between overall UPF intake and risk of developing cognitive impairment [28]. In a cross-sectional study among 2,713 older adults (≥ 60 years) from the US National Health and Nutrition Examination Survey 2011–14, UPF intake was not associated with cognitive function, except for a poorer performance in the animal fluency test among a subgroup of older adults without pre-existing conditions [27]. In contrast, other studies [26, 29–31] have reported associations between UPF intake and cognitive outcomes over time. In Brazilian adults aged ≥ 50 years (n = 10,775), higher UPF intake was associated with a faster decline in executive function and, among participants younger than 60 years only, global cognitive decline [26]. Among U.S. adults aged ≥ 60 years (n = 1375), higher UPF intake was associated with increased Alzheimer’s disease risk in those younger than 68 years, but not in older participants, and no associations were observed for dementia overall [31]. In a large UK cohort aged ≥ 55 years from the UK Biobank Study (n = 72,083), higher UPF intake was associated with increased risk of all-cause and vascular dementia, but not Alzheimer’s disease [29]. Finally, a U.S. cohort of Black and White adults aged ≥ 45 years (n = 14,175) found higher UPF intake was associated with increased risk of cognitive impairment [30].

The discrepancy between our findings and those of previous studies reporting significant associations between UPF intake and cognitive outcomes [26, 29–31] may partly reflect differences in sample size. Positive associations were generally observed in larger cohorts (n > 10,000) [26, 29, 31] providing more statistical power to detect an association than our study and other studies reporting no association [23, 27, 28]. Still, prior analyses in the same Dutch cohort did detect associations between healthy dietary patterns and cognitive outcomes [62], suggesting that sample size alone does not explain the absence of associations with UPF. Differences in dietary assessment methods may also play a role [10], but studies reporting no association—including ours in the Netherlands based on an FFQ—have used both FFQs [23, 28] and 24 h dietary recalls [27], while studies reporting significant associations also applied both methods (FFQ: [26, 30, 31]; 24-h recalls: [29]). Also, the method used to express UPF intake varies across studies, and also no clear pattern emerges. Studies reporting no association used either servings/day [23, 28] or energy ratio [27] whereas studies reporting associations used energy ratio [26], weight ratio [29, 30], or servings per day [31]. Notably, a previous study comparing weight ratio and energy ratio found that associations with incident cognitive impairment were slightly weaker when using the energy ratio instead of the primary weight ratio analyses, whereas associations with incident stroke became non-significant when using the energy ratio [30]. Since we observed no associations using the weight ratio, it is unlikely that an energy ratio approach would have changed our null findings. Differences in (type of) UPF consumption between countries may also contribute [10]. Such cross-country differences likely reflect variations in the types and nutritional composition of UPF consumed, which may potentially lead to inconsistencies in associations with cognitive outcomes. Yet, there is also no clear pattern by country: studies reporting no association—including ours in the Netherlands, come from Israel and the United States [23, 27, 28] and studies reporting significant associations are from Brazil, the United Kingdom, and the United States as well [26, 29–31]. Age may also be relevant, since UPF intake is lower in older adults [10] and some studies found stronger associations in younger compared to older adults [26, 31]. Although exposure in older adults might be insufficient or overshadowed by other known risk factors [4] the mean UPF intake in the highest quartile of our study is still substantial (33.3% ± 8.2) and suggests low exposure alone is unlikely to explain our null findings.

Another possible explanation is that overall dietary quality is more important for cognitive health than the degree of processing of the diet. Dietary intake is not included among the 14 dementia risk factors identified by the Lancet Commission due to limited evidence for a direct link [4]. However, adherence to healthy dietary patterns such as the Mediterranean diet has consistently been associated with better cognitive outcomes over time [6–8]. In line with this, our previous analyses in the same cohort found that higher adherence to the EAT-Lancet reference diet and other diet quality indices were associated with less cognitive decline [62]. The absence of an association with UPF in this cohort therefore suggests that diet quality may be more important to prevent cognitive decline than processing level per se. This interpretation also relates to the ongoing debate about the NOVA classification system. Critics argue that its broad categories hinder robust food classification and may limit the reliability of epidemiological findings [63]. Moreover, NOVA has been criticized for failing to distinguish between nutritionally poor UPFs and those with more favorable profiles, underscoring the heterogeneity of foods captured within this category [64]. While exploring the nutritional composition of individual UPFs could be interesting for future research, our analyses already adjusted for overall diet quality, which captures much of the variation in nutritional quality across foods. Nevertheless, we acknowledge that UPFs differ widely in their nutritional value—some may be relatively nutrient-dense while others are poor in nutrients—and future studies could explore whether associations with health outcomes differ across UPFs with different nutritional profiles.

This study has some strengths, including the nationally representative sample of Dutch older adults, the relatively large sample size, the investigation of multiple cognitive domains and the prospective cohort design with up to 10 years of follow-up for cognitive function and a wide range of time-dependent covariates. Moreover, dietary intake was assessed using a validated FFQ [35]. In addition, a sensitivity analysis excluding bread was performed to assess the robustness of the findings excluding a relative “healthy” UPF food group. Several limitations should also be acknowledged. Dietary intake was self-reported using an FFQ, which is prone to recall bias. Furthermore, food lists in an FFQ cannot cover all the food items consumed, which may have led to underreporting of foods including UPF [10]. In addition, the FFQ we used covered only the four weeks preceding assessment, potentially not fully capturing seasonal variation. Yet, FFQs generally have reasonable reproducibility for ranking habitual intake, even over short reference periods [65]. Finally, dietary intake was only assessed once, assuming that the intake was stable over time. These limitations would most likely have resulted in non-differential misclassification, potentially biasing the associations toward the null.

In conclusion, the findings of this study indicate that total UPF intake is not associated with cognitive function and decline among Dutch older adults. These results suggest that the level of food processing may not play a major role in cognitive aging.

## Data Availability

The data that support the findings of this study are from the Longitudinal Aging Study Amsterdam (LASA). Due to privacy regulations, the data are not publicly available but can be obtained upon reasonable request and with permission from the LASA Steering Group (www.lasa-vu.nl).
